# Testing the Assumptions in the Process of Cultural Competence in the Delivery of Healthcare Services Using Empirical Data, Focusing on Cultural Awareness

**DOI:** 10.1177/10436596231152212

**Published:** 2023-02-09

**Authors:** Nasrin Sahamkhadam, Anna Karin Andersson, Marie Golsäter, Maria Harder, Mats Granlund, Emmie Wahlström

**Affiliations:** 1 School of Education and Communication, Jönköping University, Sweden; 2 School of Health and Welfare, Jönköping University, Jönköping, Sweden; 3 School of Health, Care and Social Welfare, Mälardalen University, Västerås, Sweden; 4 Child Health Services and Futurum, Region Jönköping County, Jönköping, Sweden; 5 Child Health Care Services, Region Västmanland, Västerås, Sweden

**Keywords:** PCCDHS model, cultural competence, school nurses, children of foreign origin, Swedish school health services

## Abstract

**Introduction:**

Encounters with children of foreign origin call for school nurses’ cultural competence during the health visits. This study aimed to investigate the statistical associations between the cultural constructs described by the Process of Cultural Competence in the Delivery of Healthcare Services (PCCDHS) model and whether school nurses’ cultural encounters, cultural knowledge, and cultural skill could statistically predict their cultural awareness.

**Methodology:**

Spearman correlation and hierarchical regression analyses were conducted using cross-sectional secondary data from 816 Swedish school nurses. The cultural constructs in the theoretical description of the PCCDHS model guided the selection and sorting of the items on cultural competence.

**Results:**

The constructs of cultural knowledge, cultural skill, cultural encounters, and cultural awareness were positively correlated with each other. However, becoming culturally aware was not statistically predicted by included cultural constructs (*R*^2^ = 13.4, *p* = .06).

**Discussion:**

Despite the interrelations between the investigated cultural constructs of the PCCDHS model, understanding cultural awareness development requires further empirical testing.

## Introduction

The number of international migrants across the globe has increased over the past years, bringing about more culturally diverse populations. Children have constituted 13.9% of the total estimated population of international migrants (International Organization for Migration [IOM], 2019). Similarly, the population of children in Sweden has become increasingly culturally diverse over time. Of 2,000,000 children below 18 years in 2016, children being born abroad or having two parents both born abroad accounted for 23%, indicating a total number of 486,000 children (Statistics Sweden, 2017). These children are more likely to experience disparities related to socioeconomic status (Statistics Sweden, 2020), health status, and health needs (Gustafsson & Österberg, 2018; Hjern & Kling, 2019). Cultural competence is a response to the growing need to reduce health disparities in societies with culturally diverse populations (Abubakar et al., 2018; Eken et al., 2021). School nurses, who encounter the children on a regular basis (Michaud et al., 2021) and contribute to children’s health (Bohnenkamp et al., 2019; Musliu et al., 2019), assess themselves as moderately culturally competent (Wahlström et al., 2020). However, they also express concerns over their cultural competency when encountering children of diverse cultures (Leinander & Olsson, 2019; Musliu et al., 2019). By enhancing their cultural competence, school nurses can improve the effectiveness of their care and practices to meet the health needs of all children (Musliu et al., 2019).

Cultural competence is a theoretical concept and is defined as an ongoing process that encourages health professionals to strive to improve their personal and professional capacities in adjusting the clinical practices congruently with the clients’ cultural context in sensitive and effective ways (Campinha-Bacote, 1999, 2002; Henderson et al., 2018; Liu et al., 2021). Several theoretical models of cultural competence have been developed to explain the concept, but few have been tested empirically (Alizadeh & Chavan, 2016). Rather, studies of cultural competence have mainly focused on nurses’ levels of cultural competence and various personal and demographic factors (Ahn, 2017; Almutairi et al., 2017; Červený et al., 2020; Chen et al., 2018; Khongsamai & Intarakamhang, 2020; Ma et al., 2020; Suk et al., 2018; Wahlström et al., 2020). In the Swedish context, one particular theoretical model of cultural competence has mainly attracted nursing researchers’ attention; it is the Process of Cultural Competence in the Delivery of Healthcare Services (PCCDHS) by Campinha-Bacote (2002, 2011). This model has been applied as a framework to assess the cultural competence level in various Swedish nursing contexts with culturally diverse populations (Skoog, 2022; Wahlström et al., 2020). The model is suggested as a comprehensive, feasible framework to incorporate cultural competence as a theoretical concept into clinical practices of health professionals (Albougami et al., 2016) and could provide both theoretical and practical applications.

### The PCCDHS Model

Campinha-Bacote (2002, 2011) described the process of becoming culturally competent as represented by the theoretical model of PCCDHS that includes five cultural constructs: cultural knowledge, cultural skill, cultural encounters, cultural awareness, and cultural desire. Cultural knowledge refers to a foundation of knowledge on culturally diverse groups’ varying health and well-being. Cultural skill is the health professionals’ ability to carry out culturally adjusted health and medical assessments. The concept of cultural encounters refers to encouraging health professionals to interact with culturally diverse groups by using verbal and nonverbal communication and working with formally trained interpreters. Cultural awareness refers to in-depth self-exploration and critical reflection on one’s preconceived notions about other cultures. Cultural awareness is fundamental for identifying personal prejudices and assumptions (Andrews et al., 2010). Cultural desire is defined as the health professional’s willingness to interact with clients of diverse cultures and to become culturally competent. To develop cultural competence, all cultural constructs are equally important (Campinha-Bacote, 2002, 2011). Still, frequent cultural encounters are fundamental for the development of other cultural competence constructs. Cultural encounters may facilitate the development of health care professionals’ knowledge on cultural variations and characteristics of culturally diverse groups. In addition, cultural encounters can help health professionals to put their knowledge into practice and assess their skills in collecting relevant health data and performing more accurate assessments in the cultural context of their clients. While developing cultural skill, health professionals can also work on their cultural awareness, to explore and refine any biases and prejudices about culturally diverse clients. In addition, increasing knowledge about other cultural groups contributes to raising cultural awareness. And ultimately, more encounters might encourage the health professionals to work on their motivation and willingness to become culturally competent (Campinha-Bacote, 2011). Still, the five cultural constructs described by the PCCDHS are described as equal parts of the ongoing process of becoming culturally competent and are all assumed to be interrelated (Campinha-Bacote, 1999, 2002, 2011, 2018; Fitzgerald & Campinha-Bacote, 2019). Although the process of cultural competence is described by the PCCDHS model from this point of view, the process remains sparsely studied in the literature. Based on the model placing cultural encounters as the foundation for developing the other constructs, it is also of interest to clarify whether cultural encounters predict cultural awareness and whether cultural knowledge and skill contribute to predicting cultural awareness.

### Study Aim

This study aimed to clarify the theoretical PCCDHS model and empirically test the assumptions of the interrelations between the constructs within the model, by investigating the statistical associations between the cultural constructs described by the PCCDHS model and whether school nurses’ cultural encounters, cultural knowledge, and cultural skill could statistically predict their cultural awareness.

## Method

### Design

The present study comprises a secondary analysis of data from a cross-sectional study previously conducted in the school health setting (Wahlström et al., 2020).The cross-sectional study collected data using a web-based survey distributed between August 2017 and January 2018. The survey was distributed by e-mail using the EsMaker software and contained questions focusing on cultural competence, promotion of participation in health visits, and demographic characteristics. In this secondary analysis, only data on cultural competence from the entire sample of 816 school nurses were used.

### Setting

School health services in Sweden are the responsibility of the municipalities. The service is free of charge and offered to all children from the age of 6 to 18 years old. School nurses in the school health services invite children for regularly conducted health visits. The health visits include health assessments covering factors such as height and weight, and health dialogs to support children’s health and development (Socialstyrelsen & Skolverket, 2016).

### Sample Description

The sample in this secondary analysis consisted of all 816 school nurses from the cross-sectional study. A description of sample demographics is provided in (Wahlström et al., 2020). In summary, the school nurses were mostly females (98.5%), with males representing 1%, and the mean age was 51 (*SD* = 9) years. On a scale from less than once a month to several times a day, most school nurses reported encountering children being born abroad or having two parents both born abroad at least one or more times weekly (*n* = 547). Most school nurses spoke Swedish and one additional language and stated their country of origin as Sweden. In addition, a majority of the school nurses had completed a specialization within nursing, including education in adolescent and child health. Still, most school nurses reported receiving little to no education in cultural diversity at the workplace or in their nursing or specialist education.

### Instrument

Data concerning cultural competence were gathered using 29 items from the Clinical Cultural Competency Questionnaire (CCCTQ-PRE; Krajic & Straßmayr, 2004; Krajic et al., 2005), modified to fit the school health setting. The items were distributed over the original four subscales of cultural knowledge (six items), cultural skill (nine items), comfort level in encountering children of foreign origin (eight items), and cultural awareness (six items). All subscales were measured on a 5-point Likert-type scale. The internal consistency was estimated at α = .935 for the cultural competence scale and .83 to .89 for the subscales. In addition, an exploratory factor analysis was performed (Wahlström et al., 2020).

For the secondary analysis, the 29 items from the CCCTQ-PRE and their distribution over the four subscales were scrutinized in relation to the PCCDHS model (Campinha-Bacote, 1999, 2002, 2011) and assessed in relation to the results of the previous factor analysis (Wahlström et al, 2020). This review resulted in some of the items being sorted into other subscales to better fit the constructs of the theoretical model and the results of the factor analysis. Cronbach’s alphas for the re-sorted subscales were calculated and showed for cultural knowledge α = .85, for cultural skill α = .92, for cultural encounters α = .82, and for cultural awareness α = .72. The web-based version (Wahlström et al, 2020) and the version with new sorting of items are presented in the Supplemental Appendix.

### Data Analysis

The limit for statistical significance was set to ≤0.05. All statistical analyses were conducted using the software IBM SPSS 26 Statistics (IBM SPSS). Missing values were handled by listwise deletion; thus, observations with a missing value were dropped from the analysis (Field, 2017). Descriptive analysis was carried out to describe the distribution of responses to cultural competency subscales among school nurses. The sum of item scores for each domain was calculated. The descriptive analysis was reported as mean, standard deviation, and maximum scores for all cultural subscales. Skewness and kurtosis of the distribution of scores were calculated. The associations between the subscales were assessed by applying Spearman correlation analysis. A one-tailed correlation test was chosen as the correlations between the constructs were assumed to be in the positive direction (Field, 2017). Hierarchical linear regression analysis was carried out to identify factors predicting cultural awareness. The hierarchical regression analysis included three models based on the process described by the model (Campinha-Bacote, 2011). In the models, cultural awareness was entered as an outcome and three sets of predictors were entered: cultural encounters (Model 1), cultural encounters and cultural knowledge (Model 2), and cultural encounters, cultural knowledge, and cultural skill (Model 3). Multicollinearity in the data was assessed. The variance inflation factor (VIF) values were 2.775, 1.714, and 2.881 for cultural encounters, cultural knowledge, and cultural skill, respectively. Regarding tolerance statistics, values found were greater than 0.2, representing no collinearity in the data. Average VIF (2.456) was less than 5, indicating no possible bias in the model. The residual statistics were found to be in line with the assumption that 95% of cases had standardized residuals. More precisely, the analysis of case-wise diagnostics showed that 34 cases (4%) had extreme values and no case was found with standardized residuals greater than 3.

## Results

The distribution of data within the subscales of cultural knowledge (CK), cultural skill (CS), cultural encounters (CE), and cultural awareness (CA) showed skewness and kurtosis, see Table 1. In addition, the mean values of the subscales were closer to the maximum of the scale, especially for cultural awareness, illustrating that school nurses’ self-assessments showed moderate to high levels of CK, CS, CE, and CA.

**Table 1.** Descriptive Statistics of Cultural Competence Constructs Assessed by School Nurses.

Subscale	Max. score	*M* (*SD*)	Skewness	Kurtosis
Cultural knowledge (*n* = 813)	20	13.63 (2.98)	–0.325	0.116
Cultural skill (*n* = 813)	50	31.86 (7.46)	–0.265	–0.066
Cultural encounters (*n* = 814)	30	22.25 (3.86)	–0.509	0.648
Cultural awareness (*n* = 814)	20	16.89 (2.56)	–1.716	4.703

The results of the correlation analysis showed all subscales were related to each other, see Table 2. This supported the theoretical assumptions in the PCCDHS of all constructs being related and that increases in one construct were related to increases in the other constructs. The results also indicated that the relationship between the constructs varied, from a stronger correlation between CE and CS ( $r_{s}$ = .784, *p* = .000) to a weaker correlation between CA and all other constructs (from $r_{s}$ = .316 to $r_{s}$ = .365, *p* = .000).

**Table 2.** Spearman’s Correlation Analysis of Cultural Competence Constructs.

Subscale *N*= 812	** $r_{s}$ **			
	Cultural knowledge	Cultural skill	Cultural encounters	Cultural awareness
Cultural knowledge	1.000			
Cultural skill	.613**	1.000		
Cultural encounters	.610**	.784**	1.000	
Cultural awareness	.316**	.336**	.365**	1.000

$r_{s}$ = Spearman’s ρ coefficient.

** Significance is at the 0.01 level (one-tailed).

Statistically significant results were shown for both Model 1 (CE) explaining 12.3% of the variation in CA (*p* = .01) and Model 2 (CE and CK) explaining 13% of the variation in CA (*p* = .012). In addition, the first model only including CE in the regression also showed a better prediction ability (*F*-ratio: 114.077, *p* = .000) than the two other models (Model 2: *F*-ratio: 60.562, *p* = .000; Model 3: *F*-ratio: 41.683, *p* = .000). Hence, as described in the theoretical model of cultural competence PCCDHS, CE could be considered as having an impact on CA. Furthermore, the results indicated that including CK and CS in addition to CE in the process of becoming culturally aware did not contribute with statistical significance to explain the variation in school nurses’ CA. The results of the hierarchical regression analysis showed that 13.4% of the variation in school nurses’ self-assessed levels of CA was predicted by their self-assessed levels of CE, CK, and CS, see Model 3 in Table 3. This result was not statistically significant (*p* = .06).

**Table 3.** Hierarchical Regression Model of Cultural Competence Constructs.

Variables in model	Cultural awareness				
	*B* (Bca CI)	*SE B*	β	$R^{2}$	*p*
Model 1				.123	.001*
Cultural encounters	.236 [.183, .291]	.027	.351		
Model 2				.130	.012*
Cultural encounters	.194 [.126, .259]	.034	.290		
Cultural knowledge	.088 [.009, .177]	.039	.103		
Model 3				.134	.060
Cultural encounters	.149 [.076, .221]	.040	.222		
Cultural knowledge	.068 [–.014, .164]	.044	.079		
Cultural skill	.036 [–.006, .082]	.022	.105		

*Note. B* = unstandardized coefficient; β = standardized coefficient; Bca CI = bias-corrected and accelerated bootstrap confidence interval at 95% level.

* Significance is at the < .05 level.

## Discussion

This study showed that the cultural constructs of the PCCDHS model are statistically associated with each other, and that CE statistically predicts some of the variation in CA. The results also showed that developing CA was not statistically predicted by all other cultural constructs. Thus, the findings illustrate two assumptions: (a) the model is applicable when assuming that all constructs are positively correlated with each other, and (b) the theoretical assumption of CE as a foundation for developing cultural awareness is not applicable to school nurses in the Swedish context.

### The Relationship Between the Cultural Constructs

The findings in this study show that all four constructs are positively correlated with each other, confirming the description of relationships between the constructs in the PCCDHS (Campinha-Bacote, 2002, 2011) and related work by Campinha-Bacote (Campinha-Bacote, 2018; Fitzgerald & Campinha-Bacote, 2019). Interpreted according to the PCCDHS, the results in this study thereby indicate a positive relationship between CE and CK. Interpreted in relation to this study, CE refers to school nurses’ comfort when working with interpreters and dealing with issues related to verbal or nonverbal communication styles during encounters with children and families of foreign origin. Thereby, the results show that an increase in CE is related to an increase in CK, which refers to school nurses’ knowledge about the presence of these children at the school, the children’s health characteristics, and possible health risks. Furthermore, the results also imply that an increase in CE might improve abilities in adjusting health visits and conducting culturally based assessments (i.e., CS) as well as increase school nurses’ awareness of their biases and prejudices about children of foreign origin (i.e., CA). Still, the results in this study also show that all constructs are not as strongly correlated to each other. Among the constructs, the strongest correlations were found between CE and CS as well as CE and CK, whereas correlations between CA and the other cultural competence constructs were found to be weaker. Similar results are shown by previous research regarding strong relationships between CE and CK and between CE and CS (Siswadi et al., 2018) as well as weaker relationships between CA and other cultural constructs (Domenech Rodríguez et al., 2022; Drame et al., 2021; Eche & Aronowitz, 2017; Wahlström et al., 2020). Thus, it can be concluded that the assumption of interlinks between the cultural constructs described by the model is empirically supported by our study and previous studies, indicating that developing one competency might contribute to the development of other competencies, however with varied associations.

### Using CE as the Foundation of CA

The empirical test of the PCCDHS model reported in this study showed that CE predicts 12.3% of the variation in CA among school nurses. The theoretical description of the PCCDHS model emphasizes CE as a foundation, based on which the other constructs are continuously developed (Campinha-Bacote, 2011). Although the PCCDHS model and previous research stating the importance of CE as a basis for CA (Campinha-Bacote, 2011; Hultsjö et al., 2019; Sharifi et al., 2019), the results of this study indicate that CE might be empirically related to a small but significant percentage of the variation in CA. That 12.3% of the variance in CA is statistically explained by CE thus highlights that there might be other significant predicting factors. In addition, the small but significant prediction by CE also questions the theoretical emphasis placed on CE as a foundation for developing CA. Hultsjö et al. (2019) and Sharifi et al. (2019) also reported that encounters may contribute to the improvement of cultural awareness. Nevertheless, the study by Wahlström et al. (2020) found no statistical association between frequent encounters with children of foreign origin and the level of CA of school nurses. Furthermore, the results also show that the addition of CK and CS into the model of prediction of CA contributes to a small, but not statistically significant, increase in explained variation in CA (1.1%). Although developing CA is theoretically argued to require the development of other cultural constructs (Campinha-Bacote, 2011) and can empirically be predicted by other cultural dimensions including cultural knowledge (i.e., Reimann et al., 2004), our empirical study did not support this theoretical assumption. An explanation for this might be that school nurses need to develop their CK to be able to adjust their CS in their encounters, and in turn, refine their preconceived notions. In addition, school nurses assessed themselves as less knowledgeable and skilled in their encounters with children of foreign origin. As there are only a few studies that have been undertaken to empirically test cultural competence models, there is a need of further studies to validate these findings. Considering the results of this study, it might also be relevant to further investigate whether the process of becoming culturally competent could be explained empirically by including the concepts in a different order. The idea of increasing cultural awareness through focusing on encounters where cultural issues are placed at the center might also need to be revised based on critique of the concept of cultural competence and related models (Botelho & Lima, 2020). Such critique highlights that primarily focusing on the culture of the patients might lead to essentializing differences between groups of people and stereotypification as well as a disregard of the structural or social determinants of health (Drevdahl, 2018; Shepherd, 2019).

### Implications for Culturally Congruent Health Care

Empirically testing theoretical models provides further insights into their applicability in various health care contexts and shows whether these models can be empirically supported and confirmed. The main implication of this study concerns planning the interventions targeting health professionals working with culturally diverse clients. As educational interventions might be effective in changing CA (Chae et al., 2020; Sánchez-Ojeda et al., 2021), understanding how theories of the development of cultural competence and the interlinks of the constructs are supported in empirical testing is important. By enhancing the design of such interventions while considering the relationship between constructs, the cultural competence of health professionals might be increased even further. Increased cultural competence among health professionals may also contribute to reducing health disparities among children of foreign origin and providing equitable health care to all children (Doi et al., 2018).

### Strengths and Limitations

The strength of this study is that it examines the assumptions of PCCDHS among Swedish school nurses based on the arrangement of the subscales’ items being theoretically matched with the constructs in the PCCDHS model. The following limitations should be noted. First, despite rearrangement of the items, the CA items might not capture all aspects of the concept, which may result in a higher score on the CA subscale. Second, the CA was explained by a slight proportion of variance; thus, other factors might be related to CA and may need to be assessed. Third, the role of cultural desire should also be examined while testing the model as it might imply the development of other competencies among health professionals. Fourth, the study sample demographics should be considered when the results are interpreted and generalized.

## Conclusions

The findings of the current study contribute to a further understanding of the PCCDHS model. The results of positive correlations between the constructs, based on empirical data from Swedish school nurses, could be interpreted as statistically confirming the theoretical description of positive interrelationships between the constructs in PCCDHS. However, based on the same data, theoretically applying CE as the foundation for becoming CA could be questioned. Further investigations of what predicts CA are required to support theoretical advancements within the field of cultural competence.

## Supplemental Material

sj-docx-1-tcn-10.1177_10436596231152212 – Supplemental material for Testing the Assumptions in the Process of Cultural Competence in the Delivery of Healthcare Services Using Empirical Data, Focusing on Cultural Awareness Click here for additional data file. Supplemental material, sj-docx-1-tcn-10.1177_10436596231152212 for Testing the Assumptions in the Process of Cultural Competence in the Delivery of Healthcare Services Using Empirical Data, Focusing on Cultural Awareness by Nasrin Sahamkhadam, Anna Karin Andersson, Marie Golsäter, Maria Harder, Mats Granlund and Emmie Wahlström in Journal of Transcultural Nursing
