# Supplementary material for: Testing the Assumptions in the Process of Cultural Competence in the Delivery of Healthcare Services Using Empirical Data, Focusing on Cultural Awareness
Source: J Transcult Nurs. 2023 Feb 9;34(3):187–94. doi: 10.1177/10436596231152212 (PMC10114250; doi:10.1177/10436596231152212)
Supplement: sj-docx-1-tcn-10.1177_10436596231152212 – Supplemental material for Testing the Assumptions in the Process of Cultural Competence in the Delivery of Healthcare Services Using Empirical Data, Focusing on Cultural Awareness [file sj-docx-1-tcn-10.1177_10436596231152212.docx]

| ***Adapted CCCTQ-PRE Administrated to School Nurses*** (XX, 20XX) | ***Newly Sorted Items of the CCCTQ-PRE According to the PCCDHS Model*** (present study) |
| --- | --- |
| **Cultural Knowledge** | |
| Q1. How is your level of knowledge regarding the presence of varying ethnic groups in your school?  Q2. How is your level of knowledge regarding the cultural characteristics of varying ethnic groups?  Q3. What is your level of knowledge regarding health risks among varying ethnic groups?  Q4. How is your level of knowledge regarding health differences among varying ethnic groups?  Q5. What is your level of knowledge regarding cultural aspects of the general program in school health services?  Q6. What is your level of knowledge regarding national guidelines regarding cultural diversity in school health services? | Q1. How is your level of knowledge regarding the presence of varying ethnic groups in your school?  Q2. How is your level of knowledge regarding the cultural characteristics of varying ethnic groups?  Q3. What is your level of knowledge regarding health risks among varying ethnic groups?  Q4. How is your level of knowledge regarding health differences among varying ethnic groups?  Note: Items Q5 and Q6 were re-sorted as these items are more related to the knowledge of school nurses regarding cultural aspects of school health national programs and national guideline rather than assessing the knowledge of school nurses concerning culturally diverse groups. |
| Scoring Scale: 0= not relevant, 1= very low, 2= quite low, 3= neither low nor high, 4= quite high, and 5= very high | |

| **Cultural Skill** | |
| --- | --- |
| Q7. How qualified do you feel in greeting the child in a culturally adapted way?  Q8. How qualified do you feel in greeting the family in a culturally adapted way?  Q9. How qualified do you feel in finding out about folk medicine and alternative methods?  Q10. How qualified do you feel in conducting a culturally adapted health visit?  Q11. How qualified do you feel in providing culturally adapted health advice/counseling/guidance?  Q12. How qualified do you feel in exploring the child's / family's knowledge about health?  Q13. How qualified do you feel in working with an interpreter?  Q14. How qualified do you feel in dealing with cultural aspects concerning children's health and development?  Q15. How qualified do you feel in apologizing for cultural misunderstandings or mistakes? | Q7. How qualified do you feel in greeting the child in a culturally adapted way?  Q8. How qualified do you feel in greeting the family in a culturally adapted way?  Q9. How qualified do you feel in finding out about folk medicine and alternative methods?  Q10. How qualified do you feel in conducting a culturally adapted health visit?  Q11. How qualified do you feel in providing culturally adapted health advice/counseling/guidance?  Q12. How qualified do you feel in exploring the child's / family's knowledge about health?  Q14. How qualified do you feel in dealing with cultural aspects concerning children's health and development?  Q18. How comfortable do you feel in finding out cultural beliefs that are not expressed by parents and children but that can affect the situation?  Q20. How comfortable do you feel in interpreting different cultural expressions of pain, difficulties, and suffering?  Q21. How comfortable do you feel in giving advice to children/families on how cultural habits or beliefs can affect health?  Note. Items Q13 and Q15 were re-sorted. According to the PCCDHS model, working with the interpreter (Q13) is described as part of the cultural encounters and was sorted to the cultural encounter’s subscale. Apologizing for cultural misunderstandings or mistakes (Q15) is also matched with the description of cultural encounters construct as it measures the ability of school nurses in dealing with verbal/ non-verbal communicative patterns. Three items (Q18, Q20, Q21) from the cultural encounter’s subscale were sorted to the cultural skill subscale. |
| Scoring Scale: 0= not relevant, 1= highly unqualified, 2= quite unqualified, 3= neither unqualified nor qualified, 4= quite qualified, and 5= highly qualified | |

| **Cultural Encounters** | |
| --- | --- |
| Q16. How comfortable do you feel in encounters with children with varying cultural backgrounds?  Q17. How comfortable do you feel in encounters with children with limited proficiency in Swedish?  Q18. How comfortable do you feel in finding out cultural beliefs that are not expressed by parents and children but that can affect the situation?  Q19.How comfortable do you feel in understanding special gestures that can have different meanings in different cultures?  Q20. How comfortable do you feel in interpreting different cultural expressions of pain, difficulties, and suffering?  Q21. How comfortable do you feel in giving advice to children/families on how cultural habits or beliefs can affect health?  Q22. How comfortable do you feel in giving health advice to families of foreign origin based on national guidelines?  Q23. How comfortable do you feel in encountering children/families who have other cultural eating habits? | Q13. How qualified do you feel in working with an interpreter?  Q15. How qualified do you feel in apologizing for cultural misunderstandings or mistakes?  Q16. How comfortable do you feel in encounters with children with varying cultural backgrounds?  Q17. How comfortable do you feel in encounters with children with limited proficiency in Swedish?  Q19.How comfortable do you feel in understanding special gestures that can have different meanings in different cultures?  Q23. How comfortable do you feel in encountering children/families who have other cultural eating habits?  Note. Q18, Q20, Q21 were considered to match with the description of the cultural skill and were therefore re-sorted. These items mainly measure the ability of school nurses in finding out cultural values and collecting health information that might affect children’s health as well as providing them with health advice.  Q22 was removed as the item measures the comfort level in applying the national guideline to provide advice to children of foreign origin. The guideline does not include the content/ guide of providing health advice by school nurses to children of foreign origin.  Q13, Q15 were added as these items mainly measure the encountering between school nurses and children of foreign origin with different health habits and the ability working with interpreters, language proficiency assessment, and gestures interpretation during health visits. |
| Scoring Scale: 0= not relevant, 1= very insecure, 2= quite insecure, 3= neither insecure nor secure, 4= quite secure, and 5= very secure | |

| **Cultural Awareness** | |
| --- | --- |
| Q32. How important is cultural awareness in the interaction with the child?  Q33. How important is cultural awareness in the interaction with parents?  Q34.How important is cultural awareness in the interaction with colleagues within your own profession?  Q35. How important do you think it is for health care staff to receive training in and information about cultural diversity and/or multicultural health care?  Q36. How aware are you of your own ethnic or cultural identity?  Q37.How aware are you of your own’s preconceived notions and prejudices regarding cultural background? | Q32. How important is cultural awareness in the interaction with the child?  Q33. How important is cultural awareness in the interaction with parents?  Q36. How aware are you of your own ethnic or cultural identity?  Q37.How aware are you of your own’s preconceived notions and prejudices regarding cultural background?  Note. Q34 and Q35 appeared not to match with the construct of cultural awareness and were therefore removed. These items mainly measure the importance of working with other professionals and colleagues as well as the importance of training in cultural diversity and do not measure school nurses’ awareness of preconceived notions towards children of foreign origin. |
| Scoring Scale: 0= not relevant, 1= very unimportant, 2= quite unimportant, 3= neither unimportant nor important, 4= quite important, and 5= very important | |
